# Case Report: Guselkumab treatment for sintilimab-exacerbated psoriasis in a cancer patient

**DOI:** 10.3389/fimmu.2025.1573495

**Published:** 2025-06-20

**Authors:** Jianhao Ke, Meiliang Guo, Xuan Zhao, Na Liu, Qinqin Meng, Hui Deng

**Affiliations:** Department of Dermatology, Shanghai Sixth People’s Hospital Affiliated to Shanghai Jiao Tong University School of Medicine, Shanghai, China

**Keywords:** immune checkpoint therapy, cutaneous adverse effects, psoriasis, biologics, cancer

## Abstract

Psoriasis is a chronic inflammatory skin disease associated with multisystem comorbidities and impaired mental health. The lesions are typically characterized by sharply demarcated, erythematous plaques covered with silvery scales. Treatment options include topical agents, phototherapy, systemic therapies, and biologic agents. Traditional systemic treatments are generally contraindicated in patients with cancer due to their immunosuppressive effects. Although biologics are widely used in the management of psoriasis, their safety in patients with malignancy remains insufficiently evaluated, as individuals with cancer are typically excluded from clinical trials due to concerns about cancer progression. We report the case of a 61-year-old man whose psoriasis markedly worsened following treatment with sintilimab for pulmonary metastases secondary to colon cancer. The patient was successfully treated with guselkumab, an interleukin (IL)-23 inhibitor, resulting in significant improvement in psoriasis symptoms, while the pulmonary condition remained stable during follow-up after completion of standard cancer therapy. This case highlights the potential utility of IL-23 inhibitors as safe and effective treatment options for patients with concomitant psoriasis and malignancy.

## 1 Introduction

Psoriasis is a chronic, immunke-mediated inflammatory skin disease, typically characterized by well-demarcated erythematous plaques with overlying silvery scales. It may involve multiple organ systems and can significantly affect the patient’s quality of life and psychological well-being. Psoriasis is broadly classified into chronic plaque psoriasis and less common variants, including guttate, erythrodermic, and pustular forms. The incidence is approximately equal between men and women (1). Standard treatment options include topical agents, phototherapy, systemic therapies, and biologic agents. While the exact etiology of psoriasis remains unclear, immune dysregulation plays a major contributing factor, and various environmental and genetic factors may contribute to disease exacerbation.

Immune checkpoint therapy (ICT) has revolutionized cancer treatment by offering novel strategies for tumor control. However, ICT is frequently associated with immune-related adverse events (irAEs), which, although typically manageable, can be severe or even life-threatening in some patients (2). Sintilimab is a humanized IgG4 monoclonal antibody that targets the programmed cell death receptor-1 (PD-1), blocking its interaction with ligands to inhibit T-cell apoptosis and enhance antitumor immune response of T cells (3).

The incidence of cutaneous immune-related adverse events (ciRAEs) in patients receiving ICT has been reported to range from 20% to 40% (4). These skin manifestations commonly include nonspecific maculopapular eruptions, eczema-like or psoriatic lesions, lichenoid dermatitis, xerosis, and pruritus (5). However, to date, no published reports have documented sintilimab-induced exacerbation of preexisting plaque psoriasis. This case report presents a patient with preexisting psoriasis that was aggravated by sintilimab and assesses the therapeutic efficacy of guselkumab, an interleukin (IL)-23 monoclonal antibody, in managing this condition.

## 2 Case description

A 61-year-old male patient with a 10-year history of psoriasis was admitted to our hospital on 25 January 2024 for treatment of pulmonary metastasis following colon cancer resection. The patient received combination therapy consisting of 150 mg of oxaliplatin (L-OHP) administered intravenously on day 1; 0.7 g each of calcium folinate (CF) and 5-fluorouracil (5-FU) given intravenously on day 1; and a continuous infusion of 4.0 g of 5-FU over 46 h. Additionally, 200 mg of sintilimab was administered intravenously on day 1 as immune checkpoint blockade, and 450 mg of bevacizumab was given intravenously on day 1 for antiangiogenic therapy. This regimen was administered every 3 weeks as one treatment cycle. The patient was diagnosed with mild plaque psoriasis at Shanghai Huashan Hospital in 2014, which had remained in remission with topical corticosteroids. He reported no family history of psoriasis. However, 19 days after starting ICT, the patient developed extensive erythematous and squamous lesions, initially appearing on the medial aspects of his feet and lateral lower legs, then spreading to the trunk and scalp, accompanied by thick, layered scales. These psoriasis-like lesions were confirmed by skin biopsy (S1), consistent with a diagnosis of psoriasis. Based on the severe cutaneous manifestations and their temporal association with the PD-1 inhibitor sintilimab—which is known to activate psoriasis-related immune cells such as Th17 cells—a diagnosis of PD-1 inhibitor-exacerbated psoriasis was established. To prevent further exacerbation, sintilimab was temporarily discontinued for one treatment cycle following the second injection, then reintroduced after that cycle to maintain the effectiveness of cancer therapy.

**Figure 1:**
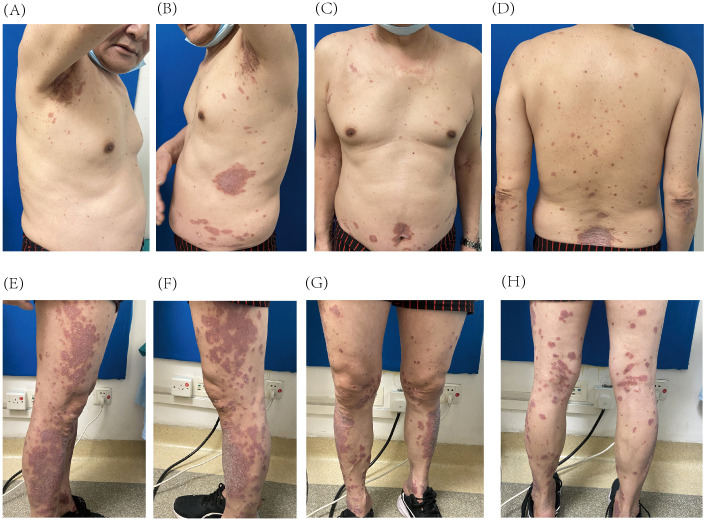
Psoriatic lesions on the trunk **(A–D)** and lower extremities **(E–H)** prior to initiation of guselkumab treatment.

The patient was referred to our dermatology clinic on 2 March 2024, presenting with a Psoriasis Area and Severity Index (PASI) score of 21.2. The skin lesions were deep red, coalescing into large patches and almost entirely covered with thick, layered scales (**Figures 1A–H**). Given the significant impact of these symptoms on the patient’s quality of life, he provided informed consent for biologic therapy after a thorough explanation of the potential risks and expected benefits.

**Figure 2:**
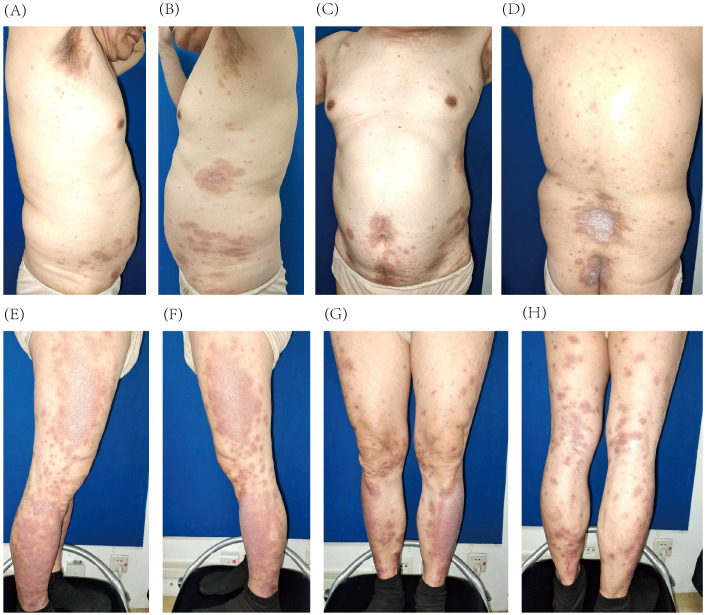
Improvement of psoriatic lesions on the trunk **(A–D)** and lower extremities **(E–H)** after 4 weeks of guselkumab treatment.

Given the patient’s history of colon cancer and evidence supporting the potential antitumor effect of IL-23 inhibitors (6–8), guselkumab was chosen based on its safety, efficacy, and tolerability profile (9). Treatment began with subcutaneous injections of 100 mg per vial of guselkumab. After 4 weeks, the patient received the second injection on 2 April 2024, resulting in a significant reduction in the number of lesions and a decrease in the PASI score to 7.2 (66% improvement). The lesions faded to a faint red with fine, subtle scales (**Figures 2A–H**). Guselkumab treatment was continued at 8-week intervals.

**Figure 3:**
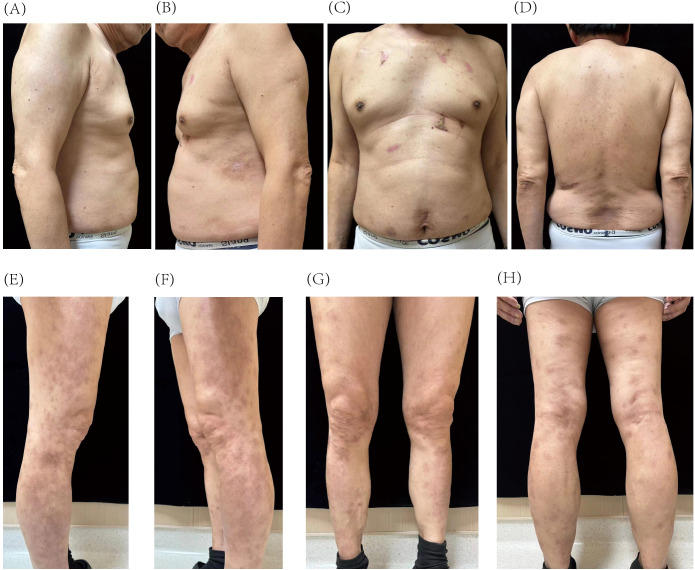
Resolution of psoriatic lesions on the trunk **(A–D)** and lower extremities **(E–H)** after 25 weeks of guselkumab treatment.

At the follow-up on 27 August 2024 (25 weeks after initial treatment), the patient achieved a PASI score of 2, representing a 90% improvement from baseline. The lesions appeared light red, with resolved skin elevation and minimal residual scaling (**Figures 3A–H**).

**Figure 4:**
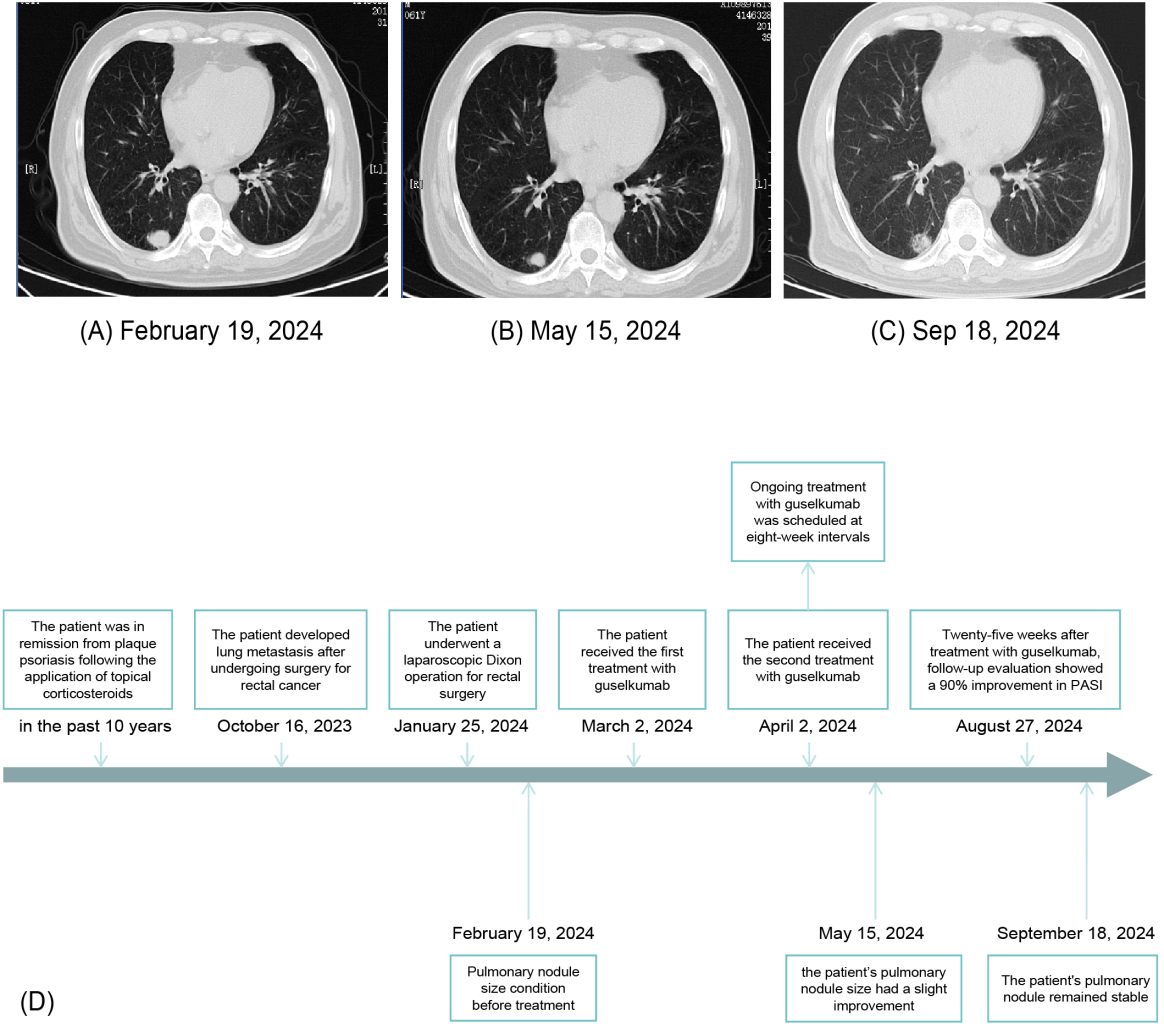
Chest computed tomography showing pulmonary nodule size before treatment **(A)**, after 10 weeks of guselkumab treatment **(B)**, and after 29 weeks of guselkumab treatment **(C)**. Timeline of the patient’s clinical course and treatment **(D)**.

Guselkumab therapy was initiated on 2 March 2024, with a second dose administered 4 weeks later, followed by maintenance dosing every 8 weeks. Liver and kidney function were monitored weekly during the first 2 months, then biweekly to monthly from months 3 to 5, and approximately every 1 to 3 months thereafter until March 2025. All test results remained within normal limits throughout the treatment period. Screening for HBV, HCV, and T-spot was negative prior to treatment initiation on 24 February 2024, and a follow-up T-spot test in November 2024 also remained negative. No signs of infection or reactivation of latent infections were observed during the treatment period, supporting the safety of guselkumab in this clinical context. The patient tolerated guselkumab well, with no adverse drug reactions reported. Despite continuing the prior chemotherapy regimen, chest computed tomography revealed a slight reduction in the size of pulmonary nodules after 10 weeks of guselkumab therapy (**Figures 4A, B**) compared with baseline imaging on 19 February 2024, indicating the safety of guselkumab in patients with concurrent cancer and severe psoriasis. At week 29, the pulmonary nodules remained stable (**Figure 4C**). These findings suggest a stable clinical response for both psoriasis and pulmonary metastasis. A timeline summarizing the clinical course was created to facilitate understanding of this case (**Figure 4D**).

## 3 Discussion

The pathogenesis of psoriasis involves the IL-17/IL-23 axis, which plays a crucial role in disease development. PD-1 inhibitor-induced psoriasis is believed to result from prolonged neutrophil activity, inhibition of macrophage apoptosis, and suppression of regulatory T-cell generation (Treg) and function. Activation and proliferation of T cells are central to the induction of psoriasis. In patients with a history of psoriasis, tissue-resident memory T cells may significantly contribute to disease recurrence following PD-1 inhibition (10–12).

Numerous biological agents targeting the IL-17/IL-23 axis have been developed for psoriasis management. The selection of appropriate biologics is crucial, especially in cancer patients. Guselkemab was preferred over IL-17 inhibitors due to its higher long-term PASI remission rates, better tolerability, and greater stability. Moreover, guselkumab offers longer dosing intervals compared to IL-17 blockers, thereby enhancing patient adherence and convenience (9). IL-17 inhibitors may negatively affect intestinal barrier homeostasis, posing risks for patients with a history of colorectal cancer and potentially leading to inflammatory bowel disease (13). Considering the patient’s history of primary rectal cancer, we deemed the use of an IL-17A inhibitor inappropriate. In contrast, treatment with IL-23 inhibitors did not induce any adverse effects. Additionally, the role of IL-17 in cancer remains controversial (14), with some studies suggesting that IL-17 inhibition may compromise the antitumor effects of ICT (15). Conversely, IL-23 inhibitors are supported by data indicating potential antitumor effects (6, 16, 17).

The antitumor properties of IL-12 and the tumor-promoting effects of IL-23 have been well demonstrated in previous studies (18). Therefore, inhibiting IL-12 could potentially compromise the body’s antitumor immune response. Additionally, safety data from a pooled analysis of briakinumab indicated that IL-12/23 inhibition in the treatment of psoriasis may be associated with an increased risk of malignancies (19). Based on these findings, we decided against this class of biologics to treat our patients.

Moreover, we opted not to use other IL-23 inhibitors due to differences in drug affinity for IL-23 and economic considerations. Among these agents, risankizumab and guselkumab exhibit approximately fivefold greater binding affinity for IL-23 and more effectively inhibit IL-23 signaling compared to ustekinumab and tildrakizumab. Both risankizumab and guselkumab fully block IL-23 binding to its receptor IL-23Rα, whereas tildrakizumab does not (20). Furthermore, guselkumab is covered by China’s national health insurance program, whereas risankizumab is not. As such, guselkumab was selected as the therapeutic option to reduce the patient’s financial burden, making it a more accessible and preferred treatment choice for similar patients in China.

Numerous studies have confirmed the involvement of IL-23 in tumor initiation, progression, and metastasis. Although its exact mechanisms remain incompletely understood, IL-23 has been shown to promote inflammation by upregulating matrix metalloprotease 9 (MMP9), enhancing angiogenesis, reducing CD8 T-cell infiltration, increasing Treg cell activity, suppressing natural killer (NK) cell perforin and interferon (IFN)-γ effector functions, and contributing to tumor persistence during the equilibrium phase (7, 8, 16, 18, 21–23).

IL-23 exerts its biological effects through the IL-23 receptor. Wight et al. demonstrated that reducing IL-23 receptor expression decreases the stability of Treg cells, thereby increasing their sensitivity to IL-12 and enhancing IFN-γ production, ultimately improving the efficiency of antitumor immune responses (6). Experimental studies have also shown that inhibition of the p40 subunit of the IL-23 receptor can activate T cells, while inhibition of the p19 subunit promotes NK cell activation (16, 17). Additionally, suppression of IL-23-mediated angiogenesis may contribute to antitumor activity (24). However, the specific effects of IL-23 inhibition depend on several factors, including the patient’s genetic profile, tumor type, and the local balance of IL-12 and IL-23. Therefore, IL-23 antibodies should be used with careful consideration of both genetic and disease-specific factors to ensure their rational application in cancer therapy (25).

Guselkumab has demonstrated a favorable long-term safety profile not only in the general psoriasis population but also in patients with a history of malignancy (9, 26). Furthermore, accumulating evidence suggests that IL-23 inhibitors may reduce the risk of various malignancies. Several studies have reported the potential effectiveness of IL-23 inhibitors against melanoma, breast carcinoma, fibrosarcoma, lung metastases, non-small cell lung cancer, non-Hodgkin lymphoma, hepatobiliary cancer, and basal cell carcinoma (7, 16, 21, 27). Nonetheless, further research is needed to elucidate the molecular mechanisms underlying these effects. While guselkumab appears to offer therapeutic benefits for patients with concurrent cancer and inflammatory disease, definitive conclusions regarding its role in oncologic therapy remain unsupported by sufficient evidence. Clinicians should therefore carefully weigh the potential risks and benefits when managing such complex clinical cases. Ongoing investigation is essential to clarify the role of IL-23 inhibitors in cancer treatment and to inform future evidence-based guidelines.

In conclusion, although sintilimab-associated psoriasis is a rare immune-related adverse event in lung cancer treatment, it can profoundly affect a patient’s quality of life and mental health. To the best of our knowledge, this is the first reported case of successful management of sintilimab-exacerbated psoriasis using guselkumab, highlighting a potential therapeutic approach for this uncommon but clinically significant complication.

## Patient perspective

I agreed to the use of guselkumab for alleviating my lesions after being fully informed by my physician of the potential risk of metastasis, as the severity of my psoriasis had caused me considerable distress. The actual therapeutic response has confirmed that guselkumab was the right choice for managing my condition.

## Data Availability

The original contributions presented in the study are included in the article/Supplementary Material. Further inquiries can be directed to the corresponding author.
